# Novel pheromone-mediated reproductive behaviour in the stag beetle, *Lucanus cervus*

**DOI:** 10.1038/s41598-024-55985-8

**Published:** 2024-03-12

**Authors:** Deborah J. Harvey, József Vuts, Antony Hooper, John C. Caulfield, Paul Finch, Christine M. Woodcock, Alan C. Gange, Jason W. Chapman, Michael A. Birkett, John A. Pickett

**Affiliations:** 1 Department of Biological Sciences, Royal Holloway University of London, Egham, TW20 0EX UK; 2 Protecting Crops and the Environment, Rothamsted Research, Harpenden, AL5 2JQ UK; 3 Centre for Ecology and Conservation, University of Exeter, Penryn Campus, Penryn, Cornwall, TR10 9FE UK; 4 School of Chemistry, Cardiff University, Cardiff, CF10 3AT UK

**Keywords:** Biochemistry, Ecology, Behavioural methods

## Abstract

The iconic European stag beetle (*Lucanus cervus*) (Coleoptera: Lucanidae) is one of the largest terrestrial beetles in Europe. Due to decreasing population numbers, thought to be a consequence of habitat loss, this beetle has become a near-threatened species across much of Europe, and a reliable monitoring system is required to measure its future population trends. As part of a programme aimed at conserving UK populations, we have investigated the chemical ecology of the beetle, with a view to developing an efficient semiochemical-based monitoring system. Such a scheme will be beneficial not only in the UK but across the European range of the species, where the beetle is of conservation concern. Here, we report on a surprising discovery of a male-produced pheromone, which provokes initial sexual receptivity in females, and which has not been previously identified in the animal kingdom. Furthermore, we assign sex pheromone function to a previously described female-specific compound.

## Introduction

The European stag beetle, *Lucanus cervus* (L.) (Coleoptera: Lucanidae), is probably one of the most easily recognised insects, particularly the male with its enlarged mandibles and large size which, in the UK, reaches up to 70 mm^1^. This species is classified as near-threatened^2^ across much of its range and is extinct in Denmark^3^. Moreover, little is known about its chemical ecology and how this knowledge could be used to develop an accurate monitoring scheme for the species.

The cryptic life cycle of *L. cervus* begins in late summer, when the female beetle lays up to 36 eggs individually and close to a subterranean deadwood source, with larval development taking up to 6 years^4^. A further six weeks are spent as a pupa, with the newly eclosed beetle remaining underground for the next nine months to emerge the following summer when temperatures exceed 16.5 °C for a prolonged period^4,5^.

In the UK at least, where the species is predominantly an urban beetle found mainly in private gardens, the trend for ‘clean’ gardens with the removal of dead wood and stumps is believed a contributing factor to population decline^6–8^. Declines across the rest of its European range, where, with the exception of Germany and Belgium, the habitat is predominantly along the edges of deciduous woodlands, are also largely believed to be due to habitat loss^2,9,10^. However, to assess accurately whether the numbers of *L. cervus* are falling, a monitoring scheme is needed to yield reliable results.

The prolonged, subterranean larval phase and comparatively short visible adult phase of the life cycle, lasting up to approximately twelve weeks^4,8,11^, have made this species extremely difficult to monitor accurately. In the UK, numerous annual citizen science-based initiatives to track the distribution of the species, e.g. The Great Stag Hunt^6,7^, have been conducted, which have relied on members of the public reporting sightings of this crepuscular beetle. Participants are encouraged to search for the adult male, which is described as typically flying on humid summer evenings at dusk. However, the tendency of males to fly in a circular path^11^ may result in overcounting, giving a false impression of the numbers. Moreover, sightings may be linked to areas where monitors are found on pleasant summer evenings, such as urban gardens, parks and public house gardens^6^.

Schemes have been established across the UK and mainland Europe to provide habitats for the species, such as the provision of artificially placed log piles and a ‘Bury Buckets for Beetles’ scheme to provide continual habitat sources. Furthermore, a European initiative has developed a protocol for monitoring the beetle using walking transects carried out by volunteers, supervised by scientists in participating countries (stagbeetlemonitoring.org)^12^. However, a non-destructive method which accurately determines the number of beetles without the active presence of monitors and does not result in the death of beetles is needed across Europe to fulfil European Habitats Directive^2,8^.

Chemical lures developed from semiochemicals released by the species in question have been widely used to attract and monitor pest insects globally^13^. However, for those working on insects of conservation interest rather than economic impact, this technique is only slowly gaining momentum^14–16^ to monitor the success of conservation measures^8^. Such a methodology is critical in non-feeding adults, such as those of *L. cervus*, where aggregation does not occur at feeding sites and therefore attraction of mates must rely on alternative means.

In many beetle species, the sexually mature females produce and release long-range sex or aggregation pheromones to attract males and initiate reproductive behaviour. Short-range insect aphrodisiac pheromones are released by males to elicit mating behaviour once both sexes are in proximity. In *L. cervus*, longifolene has been described from headspace samples of females and larvae and proposed to be responsible for male attraction to these life stages^8^, but no structure assignment and behavioural activity studies have been reported. The chemically mediated reproductive behaviour of adult *L. cervus* was therefore investigated to test the hypotheses that i) longifolene acts as a female sex pheromone, and that ii) male-emitted pheromones are produced in response to the female pheromone and promote copulation. In the future, use of at least one of these compounds could lead to an improved monitoring scheme.

## Results

### Female sample analysis

**Figure 1:**
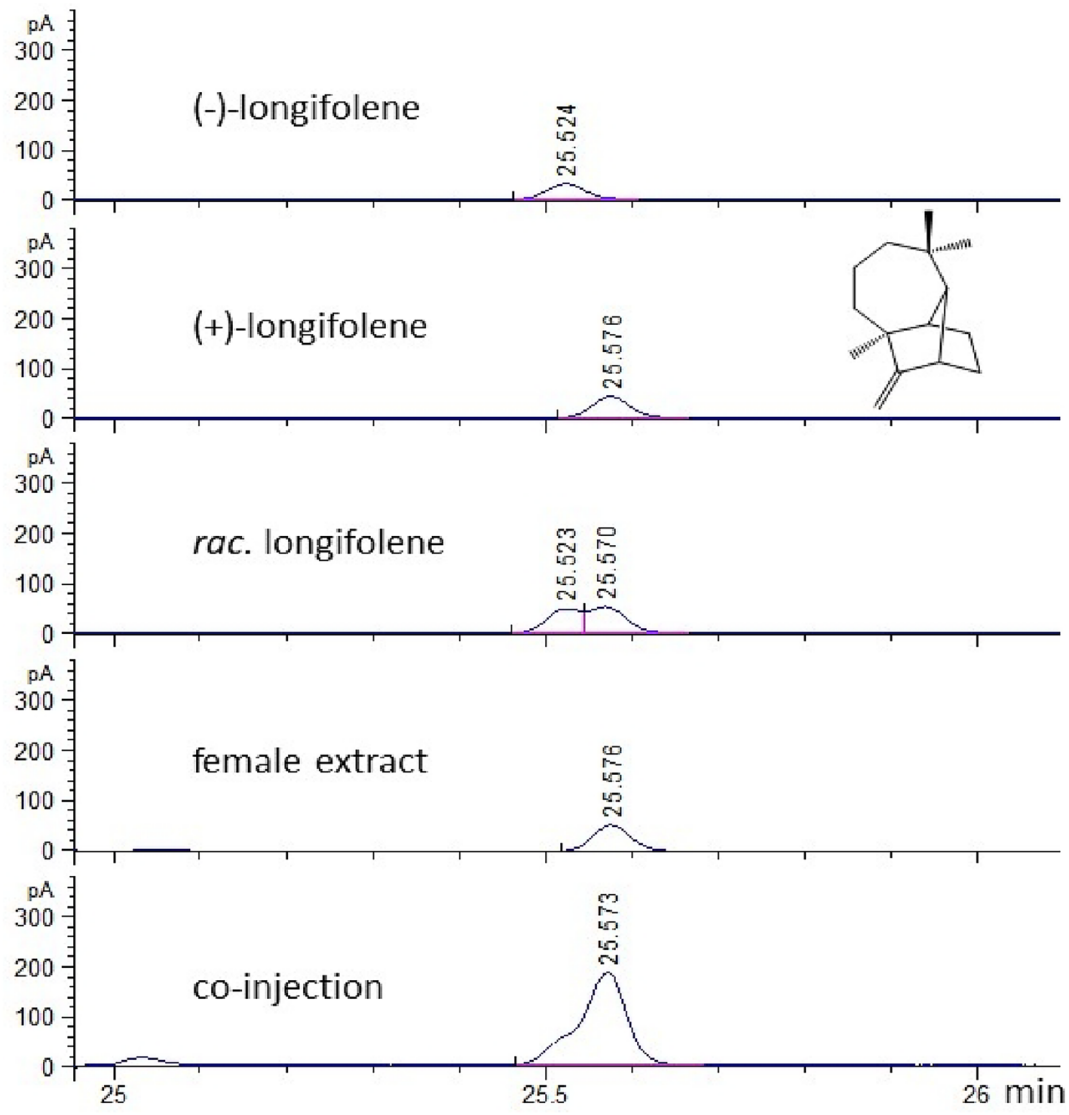
Structure confirmation of longifolene in a female *Lucanus cervus* air entrainment extract, using a chiral GC column.

**Figure 2:**
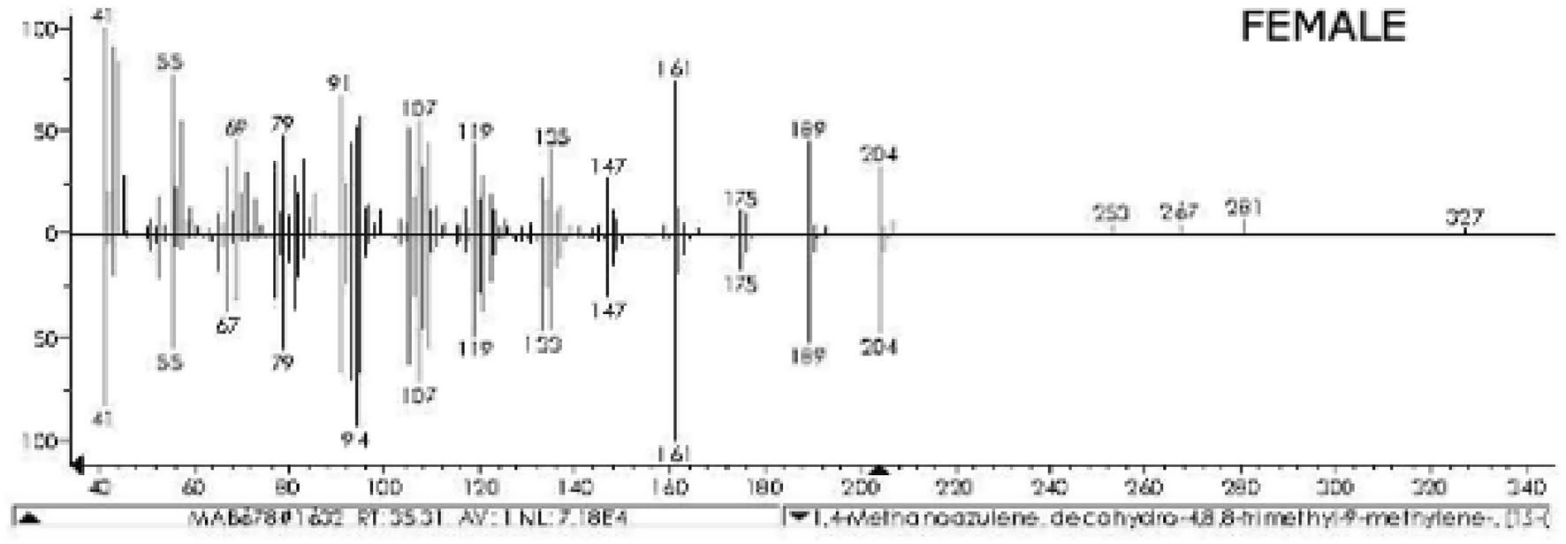
Mass spectrum of longifolene identified from female swab samples, taken from the cuticle abutting the yellow spot area (see “Methods” section and Fig. 6) on the femur of the first pair of legs, in comparison with the spectrum from the library database (lower trace).

Analysis by GC and GC–MS of air entrainment and swab samples from female beetles confirmed the presence of longifolene [KI (Kováts index) on a HP-1 apolar GC column: 1415], and further investigation on a chiral GC column identified it as (+)-longifolene (C_15_H_24_) (Figs. 1 and 2). The mean (+)-longifolene release was estimated from air entrainment samples to be 4.2 ± 1.2 ng/female/20 min. Trace amounts of ɑ-copaene and ɑ-pinene were also found in female extracts.

### Male sample analysis

**Figure 3:**
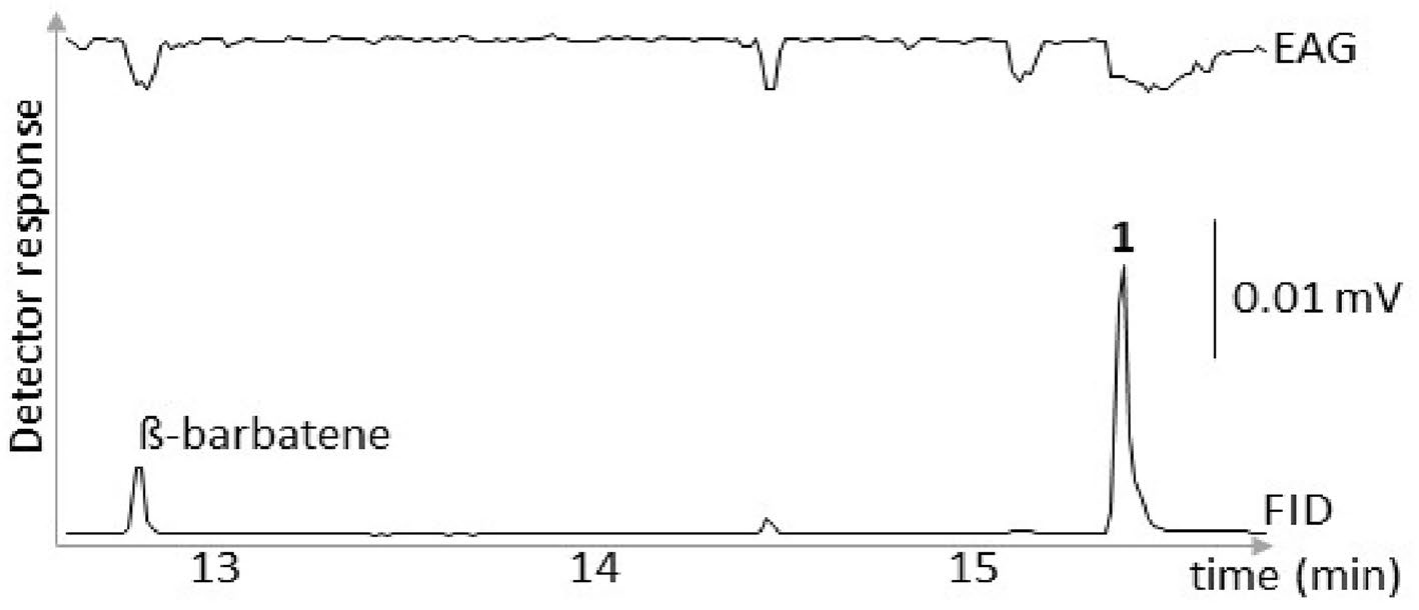
Coupled GC-EAG trace of female antennal responses to a male *Lucanus cervus* air entrainment extract. The two male-specific peaks that elicited consistent EAG activity in at least three coupled runs are labelled. As the peaks at ca. 14.30 min and just after 15 min did not evoke consistent EAG responses across the replicate runs (active in only one run), their identification was not pursued.

Initial screening of male air entrainment extracts by coupled GC-electroantennography (GC-EAG) located two bioactive peaks (Fig. 3).

**Figure 4:**
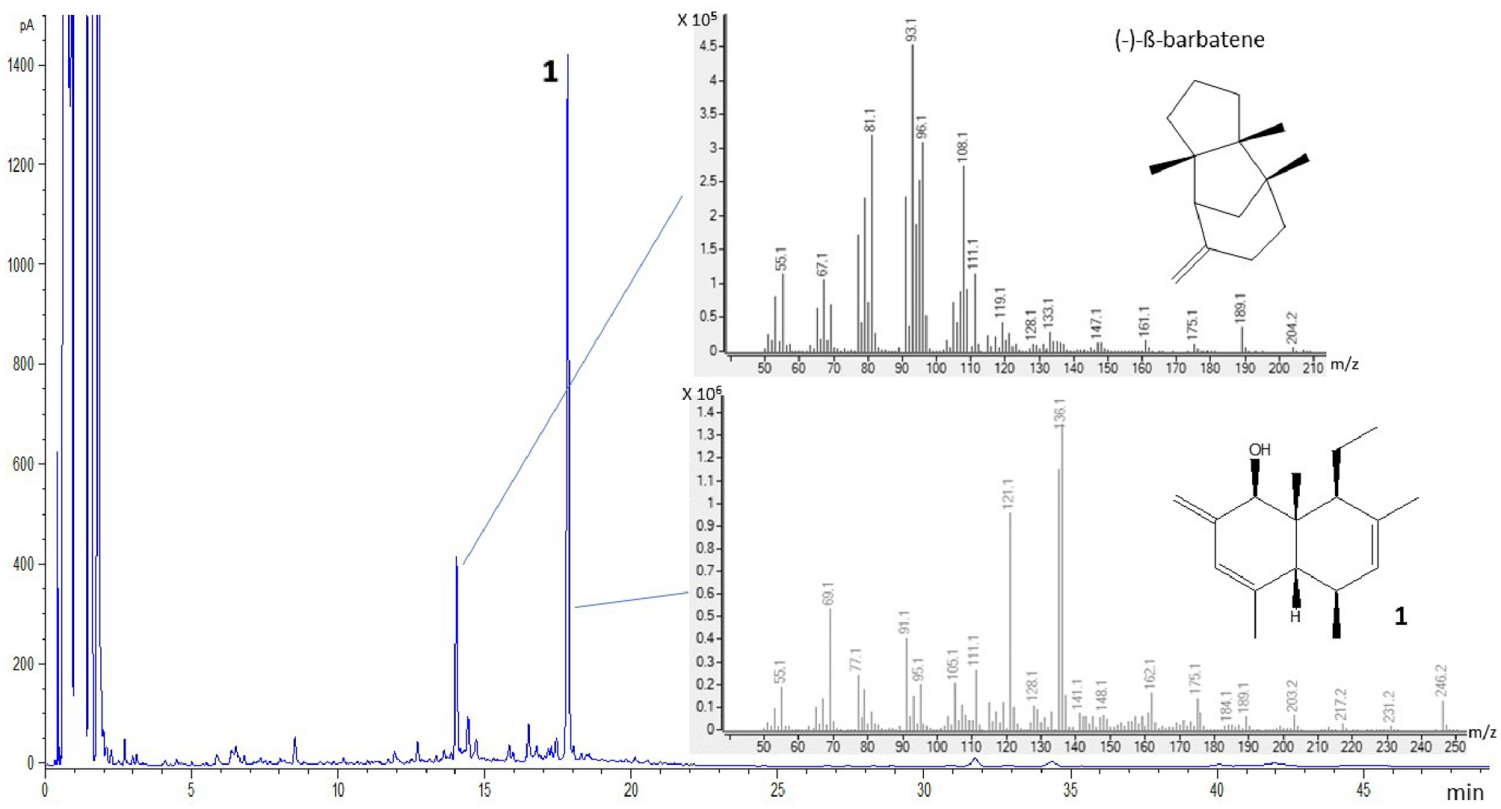
GC profile of a male *Lucanus cervus* air entrainment extract, showing the (−)-ß-barbatene peak, its mass spectrum and structure. A second male-specific peak **1** was tentatively identified with the empirical formula C_17_H_26_O with structure **1**.

The first peak had a molecular ion giving a molecular weight of 204 amu and a KI on a HP-1 apolar GC column of 1455, indicating that it was a sesquiterpene hydrocarbon (Fig. 4). The fragmentation pattern suggested it to be an isomer of ß-barbatene^17^, a chiral sesquiterpene not reported previously in the animal kingdom. A sample of the (−)-enantiomer was isolated from the liverwort *Bazzania trilobata* L. (Lepidoziaceae) (suppl. Info. S1)^18^ and used for peak enhancement by GC co-injection (mean release: 7.2 ± 1.9 ng/male/20 min).

The second male-specific compound **1** had a molecular weight of 246 amu and a KI of 1750, but a structure could not be determined from the mass fragmentation pattern (Fig. 4) without more structural information at this stage.

**Figure 5:**
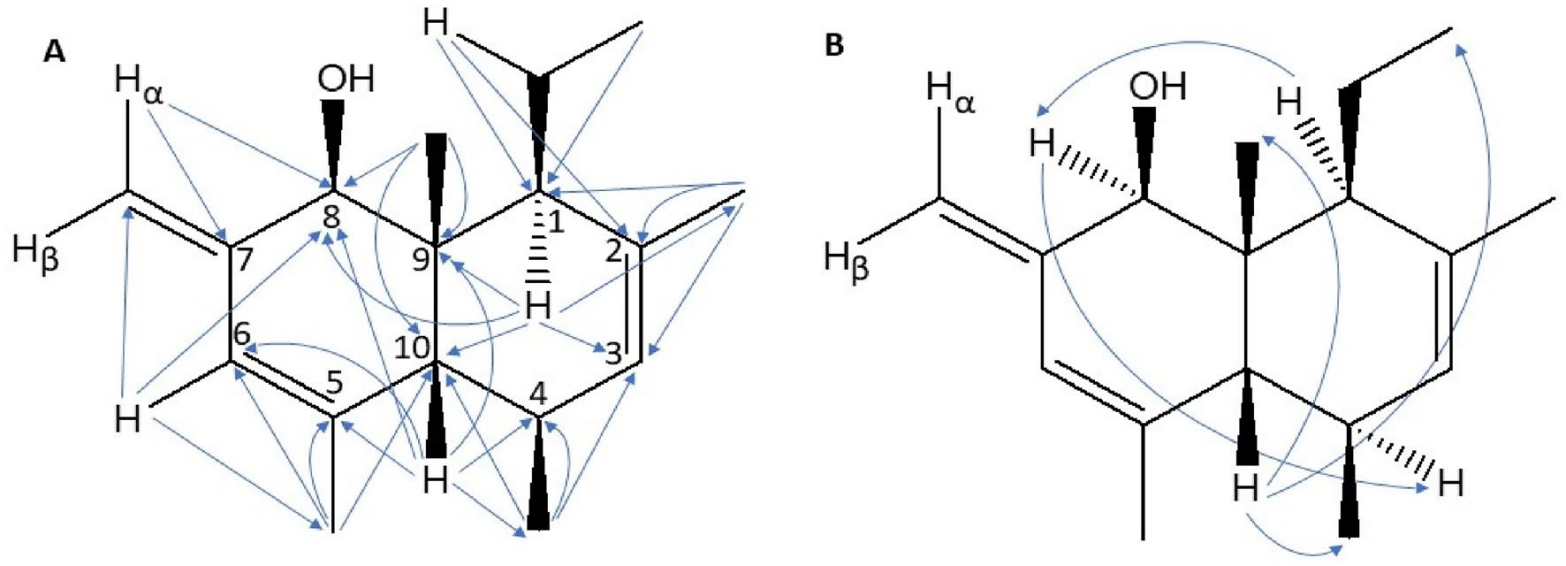
NMR analysis of a purified fraction of **1**. (**A**) Significant HMBC correlations to deduce the 2D structure. (**B**) Significant nOe correlations to deduce the 3D relative stereochemistry.

**Figure 6:**
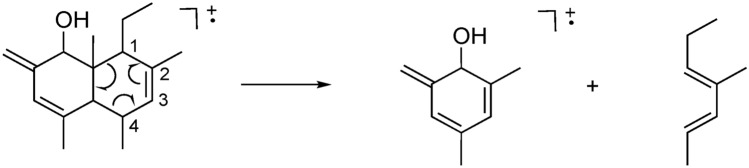
Mass spectrometer (EI) retro-Diels Alder rearrangement of the molecular ion at m/z 246, from the molecular structure** 1**, to give the radical ion at m/z 136 and the neutral fragment.

Preparative-scale GC, using a HP-1 column, resulted in the isolation of 60 μg of the unknown male compound from male headspace collections (suppl. info. S1). The structure of **1** was deduced through standard NMR processes, although accumulation of the data on such a small sample required the use of gradient pulses and a 1 mm capillary probe. All protons, apart from the exchangeable hydroxyl proton, were characterised and correlated to the carbon atom they were bonded to by gradient HSQC correlations. Through gradient proton-proton correlations and gradient HMBC correlations (Fig. 5A), the relative position of each moiety could then be assigned and all the carbon atoms characterised. Nuclear Overhauser effect (nOe) spectroscopy was performed and on the limit of resolution with such a small sample. However, nOes were observed between H-10 and the methyl protons on the 9-Me and 1-Et groups so assigning them on the same face. In addition, nOe correlation between H-1 and H-8 and between H-8 and H-4 implied these protons were on the opposite face (Fig. 5B). As a result, the structure **1** is proposed with the relative stereochemistry shown (Fig. 5B). Further accurate mass measurements in mass spectrometry by electron ionisation (EI) determined the m/z (mass to charge ratio) value of the molecular ion as 246.2045, which is consistent with the proposed structure **1** having the empirical formula C_17_H_26_O. A key ion supporting the proposed structure **1** is from a retro-Diels Alder rearrangement of the molecular ion (parent radical ion) giving the radical ion at m/z 136, corresponding to C_9_H_12_O, the most abundant ion in the spectrum (Fig. 6) through loss of the neutral fragment at C1–C4, from the molecular structure **1** (Fig. 6). The nomenclature for **1** is thus suggested as (1*R/S*,4a*R/S*,5*R/S*,8*S/R*,8a*R/S*)-8-ethyl-4,5,7,8a-tetramethyl-2-methylene-1,2,4a,5,8,8a-hexahydronaphthalen-1-ol.

The mean release of this compound was 13.6 ± 3.9 ng/male/20 min.

^1^H NMR (500 MHz, d_6_-Benzene) *δ* 5.90 (1H, s, H6), 5.34 (1H, s, H7a), 5.25 (1H, s, H3), 5.06 (1H, s, H7b), 4.56 (1H, s, H8), 2.33 (1H, dd, *J* = 6.1, 5.5 Hz, H1), 1.96 (1H, m, H4), 1.86 (3H, s, 2-Me), 1.85 (3H, s, 5-Me), 1.76 (1H, d, *J* = 7.9 Hz, H10), 1.66 (1H, m, H1a), 1.40 (1H, m, H1b), 1.14 (3H, d, *J* = 6.8 Hz, 4-Me), 1.05 (3H, t, *J* = 7.5 Hz, 1-Me), 0.94 (3H, s, 9-Me). ^13^C NMR (500 MHz, d_6_-Benzene) *δ* 141.1 (C5), 134.5 (C2), 126.8 (C3), 124.0 (C7), 123.8 (C6), 109.3 (7-CH_2_), 71.0 (C8), 50.0 (C10), 45.1 (C1), 42.0 (C9), 37.5 (C4), 29.9 (1-CH_2_), 25.0 (5-Me), 24.1 (2-Me), 23.3 (4-Me), 19.7 (1-Me), 17.2 (9-Me). EIMS *m/z* 246 (M^+^, 10), 55 (15), 69 (39), 77 (19), 91 (30), 95 (16), 105 (16), 111 (20), 121 (72), 135 (85), 136 (100), 162 (13), 175 (10), 189 (4), 203 (5), 217 (2), 231 (1).

### Authentic standards

Sesquiterpene hydrocarbon required for identification of the pheromonal compound (+)-longifolene, described in formal IUPAC nomenclature as (1*R*,2*S*,7*S*,9*S*)-3,3,7-trimethyl-8-methylenetricyclo-[5.4.0.0^2,9^]undecane, was purchased from Sigma-Aldrich UK (> 99%). The enantiomer (−)-longifolene was donated by the late Wittko Francke, University of Hamburg, and had been synthesised according to Schulz and Puig^19^.

(−)-β-Barbatene was isolated from the liverwort *Bazzania trilobata* L. (Lepidoziaceae)^18^. Fresh samples of *B. trilobata* were collected from Argyllshire, UK, air-dried, frozen with liquid nitrogen and crushed. The powder was split into two equal samples, and each was extracted with hexane at room temperature for 48 h and the solvent recovered by filtration (gravity). The extracts were evaporated under reduced pressure to yield dark green residues (3.34 and 5.78 g). The first residue was subjected to repeated liquid chromatography over Florisil using hexane as the elutant, and collected fractions were analysed by GC. Fractions enriched in single components were combined. One of the combined samples, shown by GC to be a single component (> 95% pure) comprised a colourless, free-flowing oil (220 mg), which was analysed by 500 MHz NMR. Comparison of the NMR data with the literature confirmed the identity of the oil as (−)-ß-barbatene^17^. The authentic sample was used to confirm the stereochemistry of the β-barbatene peak in a male air entrainment extract.

### Confirming area of volatile release

SEM examination at 250–1700 × magnification of the pronotal cuticle abutting the trochanter of the first pair of legs revealed an area of cuticle with a high gloss, penetrated with pores, some of which had evidence of a substance emanating from them. A patch of dense setae associated with pores at the base surrounds the area of cuticle. The setae around this area appear to be within a socket and therefore have some mobility. There is no evidence of pores within the structure of the setae either along the shaft or at the tip. The setae are therefore unlikely to be receptors but more probably involved in the distribution of the substance emanating from the pores, i.e. pheromones^29^ (Supplementary Information Fig. S2).

### Behavioural assays

#### Determination of cues for mating behaviour

When the ten males were exposed to females placed in transparent, sealed containers, none of them approached the container and attempted to access the female. However, when the males were exposed to sexually receptive females placed in open opaque boxes, all ten males walked directly towards the container with their antennae held at right angles to the head and maxillary palps showing rapid movements and the first pair of legs raised. On contact, the male lowered his body, mounted the female, extended his genitalia and investigated the female with his galea and palps. Females did not approach males when they were sealed in transparent boxes or placed in opaque containers.

#### Response to female-produced volatiles

##### Male response

When the thirty-two males were exposed to 20-uL samples taken from the yellow spot region of females, they all approached the vial and started to palpate it, but none of them responded to 20 μL of pure diethyl ether (control).

Thirty of the 32 males placed in a choice chamber individually approached the opaque container holding (+)-longifolene and palpated the vial, followed by extending their genitalia and attempting copulation (Fisher’s Exact, d.f. = 1, N = 32, P < 0.001).

##### Female response

None of the twenty females exposed to extracts from the female yellow spot area showed any visible response, neither did they show a response to the (+)-longifolene sample.

All males and females exhibited attraction to ɑ-pinene, showing antennation and movement towards the vial; however, no palpating of the vial was observed. Both sexes tested individually with ɑ-copaene in choice chambers showed similar attraction but no palpation.

#### Response to male-produced volatiles

##### Male response

Twenty-eight out of thirty-two males exposed to samples taken from the male yellow spot region demonstrated aggressive behaviour, attempting to fight with the vials (Fisher’s Exact, d.f. = 1, N = 32, P < 0.05).

Males showed no reaction to (−)-β-barbatene; however, when exposed to the second, tentatively identified male-specific volatile **1** as a purified sample at 200 ng, but not 100 ng, dose all males responded by becoming aggressive and attempting to fight with the vial.

##### Female response

When twenty females were exposed to vials containing extracts from the male yellow spot area, they did not approach it, but all took up a sexually receptive pose.

When females were placed in a choice chamber with (−)-β-barbatene, eight of the ten tested took up a sexually receptive posture (Fisher’s Exact, d.f. = 1, N = 10, P < 0.05), and when replaced in a tank of males, were approached by males and started mating.

Females, when placed together in a choice chamber with no chemical stimulus, did not display any increase in sexual receptivity described above.

##### Wind tunnel experiments

All fifteen males tested in the wind tunnel for their response to the swabs taken from the yellow spot area of females and to 20 μL (+)-longifolene moved towards the stimuli and antennated the vials in less than two minutes. None of the beetles reacted to the diethyl ether solvent control. No females were attracted to swab samples taken from the male yellow spot area, indicating that the patch of yellow setae on the female femur is a source of a long-range male attractant.

##### Aerial trap results

A total of four males were caught in traps baited with 20 μL of (+)-longifolene during one trapping season. No beetles were caught in control traps.

## Discussion

Here, we present the first evidence of sexually active pheromonal compounds produced by *L. cervus* and outline their role in initiating mating within the species. These compounds include (−)-β-barbatene not previously reported in the animal kingdom and a second, male-produced compound as a new, tentatively identified natural product. Only they elicited consistent antennal responses in coupled GC-EAG runs, thereby diverting further structural investigation. Although behaviourally active as a purified fraction of headspace extracts, synthesis of the novel male volatile for absolute identification and to demonstrate its role as a semiochemical will be necessary.

Adult *L. cervus* release volatiles from the cuticle on the pronotum opposite a patch of yellow setae on the trochanter of the first pair of legs that initiate mating behaviour and subsequent copulation. We propose the released pheromone soaks onto the setae when the beetle is at rest and the setae are in contact with the cuticle, but when it becomes sexually receptive, the beetle lifts the body away from the leg, allowing the volatiles to evaporate. Such pheromone release mechanism has been described for other lamellicorn (Scarabaeoidea) species^20^ and antlions^21^.

As seen in many species^13,22,23^, females of *L. cervus* produce a sex pheromone, identified as (+)-longifolene, which attracts conspecific males. (+)-Longifolene is also produced by larvae, along with α-copaene^8^. The production of the same volatile compounds by the immature stage and females of a species has been previously identified in *Cyclocephala lurida* chafers (Coleoptera: Scarabaeidae^24^) and suggests the evolutionary development of sex pheromones from chemicals that previously played a role in larval physiology or behaviour. The production of such chemicals is lost in adult males but retained in females and larvae because of a primary function in communication^11^.

ɑ-Copaene and α-pinene, although demonstrated to be attractants, do not elicit sexual behaviour in either sex but may enable females to locate an oviposition site^11^. Since male *L. cervus* emerge before females^4^, the attraction of males to (+)-longifolene, α-pinene and α-copaene can help them to detect the presence of female beetles prior to their emergence from under the ground, ensuring that potential mates are found and territories established before the short activity phase begins, a crucial factor in a species where the adults are non-feeding. Females are not attracted to (+)-longifolene but may use it to identify suitable oviposition sites.

On suitable evenings during the mating period (June-July) when temperatures rise above 16.5 °C, *L. cervus* males are seen flying at dusk in circular paths, returning to mate in established territories throughout the night^4^. We propose that during these flights, males are diverted from their territory by females releasing (+)-longifolene, thereby increasing the chance of mating in this polygamous species. As evidenced by the collection and analysis of swabs from both sexes of beetle^8,11^, once in the presence of a female, the male releases (−)-ß-barbatene as an aphrodisiac pheromone, which increases sexual receptivity of other females in the vicinity and induces them to release (+)-longifolene, bringing in more males. This is the first reported instance of (−)-ß-barbatene being used for sexual communication in insects; previously, it has been associated with fungi and liverworts^17,27^. The aggregation of males at mating sites may lead them to produce the second male compound, which elicits aggression in males and acts as a method of mate guarding; swab evidence indicates that males do not produce (−)-β-barbatene, nor compound 1, in the absence of females^11^. Furthermore, compound 1 was only detected in samples from males in the presence of at least another male, which subsequently had taken up the sexually receptive pose and become aggressive. Such breeding behaviour serves to increase mating opportunities in a species which has limited flight distance (maximum 1 km in males^28^) and is non-feeding as an adult.

The chemical characterisation and the determination of the role played by the compounds produced by *L. cervus* has allowed us to explore the complex chemical communication channels underlying the intricate mating system of this iconic saproxylic species. Furthermore, it may offer opportunities for the development of non-invasive, live monitoring tools, such as pheromone-baited trapping schemes that may indirectly help preserve fragmented populations of *L. cervus* across the entirety of its European range.

## Methods

Apart from where mentioned otherwise in the text, all behavioural assays were carried out in a box with dimensions 50 cm ⨯ 35 cm ⨯ 8 cm.

### Initial behavioural observations and volatile collection

**Figure 7:**
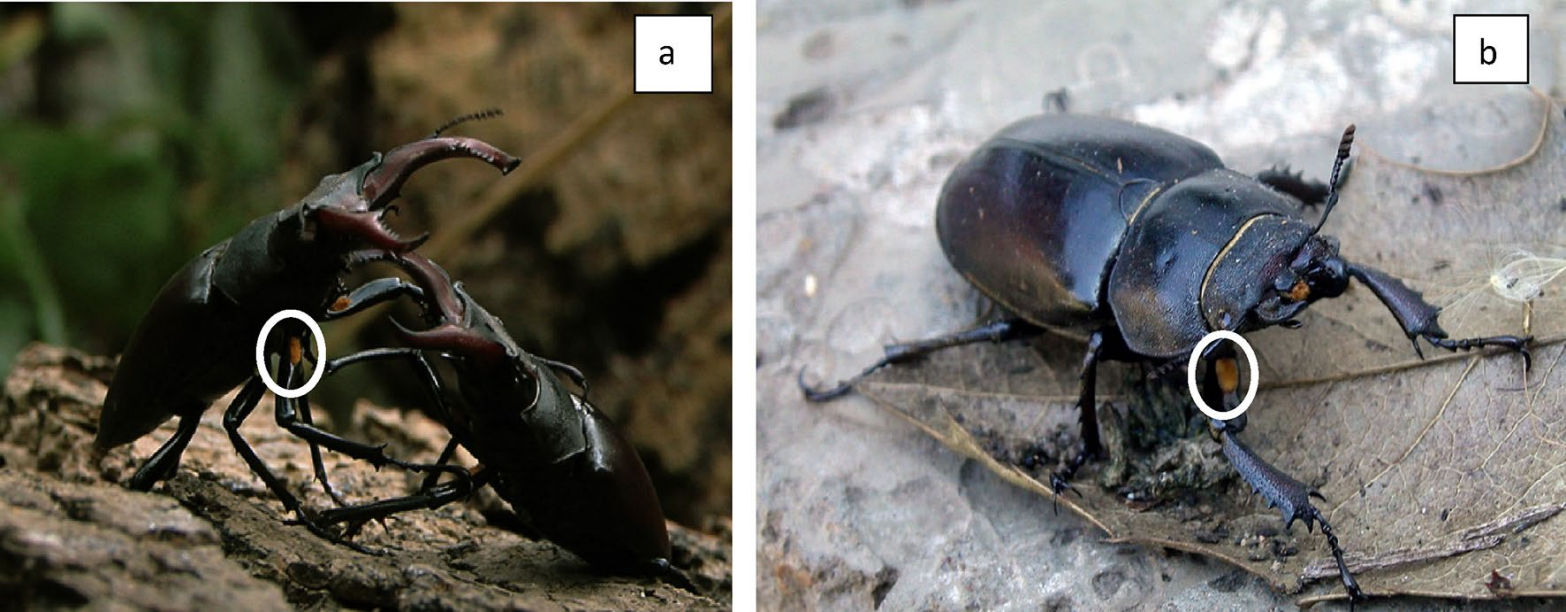
Patch of dense yellow setae on the femur of the first pair of legs in both male (**a**) and female (**b**) beetles.

A series of bioassays were carried out to determine the behaviour of beetles leading up to copulation. Ten beetles of each sex were placed into choice chambers and observed for 10 min. Prior to mating, males assume a raised ‘sexually receptive’ position (Fig. 7), lifting the thorax such that a patch of yellow setae on the femur of the first leg is made visible. They then move directly towards a female, but only if she is exhibiting the same pose (Fig. 7). A pre-mating ritual then starts, with the male and female aligned with the male uppermost and in the same orientation, the male then turns 180° and moves his genitalia across the female’s palps, whilst he palpates the female’s genitals. The male then turns to his original orientation and copulation ensues. When a mating pair was established, the beetles were removed from the tank and placed into a holding vessel.

The separated mating pairs were then swabbed using fine glass swabs (Fisher Scientific, UK) to brush the cuticle adjacent to the dense patch of setae on the femur of the first leg of both sexes of adult beetles, after which the pair were placed back together to reinitiate mating. The change of posture of both sexes prior to mating suggested that this region may be the site of pheromone production as in other beetle species^29,30^. Swabs were collected into 10 µL redistilled diethyl ether and left overnight. The resultant solution was tested for behavioural responses in choice chambers and wind tunnels, the latter to mimic field conditions. Redistilled diethyl ether was used as a control.

Dynamic headspace extraction (air entrainment) was carried out on of both sexes in glass chambers (Supplementary Information Fig. S1). The collection was performed for 22 h onto 50 mg Porapak Q polymer (50/80 mesh, Sigma-Aldrich, UK) under positive pressure of charcoal-filtered air (air pumped in at 600 mL/min and pulled out at 500 mL/min), after which time the volatiles were eluted with 750 µL redistilled diethyl ether and concentrated under a gentle stream of nitrogen gas.

### Sample analysis

Samples (2 μL) were subjected to gas chromatography (GC), followed by coupled GC-mass spectrometry (GC–MS) analysis. The GC (HP6890) was equipped with a HP-1 column (50 m ⨯ 0.32 mm ID, 0.52 μm film thickness; Agilent, Santa Clara, CA, USA) and a cool-on-column injector. A run time of 30 min was used with the column heated from 30 °C at a rate of 5 °C/min up to 150 °C, then 10 °C/min to 250 °C. Enantioselective GC work with the insect-derived natural products used an Agilent 6890 N GC equipped with a cool on-column injector, an FID and a 30 m × 0.25 mm ID × 0.25 μm film thickness SUPELCO® Beta DEX™ (Sigma-Aldrich, Gillingham, UK) 120 fused silica capillary column. The oven temperature was maintained at 30 °C for 1 min and then programmed at 5 °C/min to 150 °C, then at 10 °C/min to 230 °C and held for 22 min. The carrier gas was hydrogen.

GC–MS analysis used GC conditions as above connected to a MAT95XP mass spectrometer. The run time was 49 min, with the temperature of the column maintained at 30 °C for 5 min then ramped at 5 °C/min to 250 °C. Ionisation was by electron impact at 70 eV. Tentative identification of spectra was made by comparison with the NIST 2002 library and was confirmed using GC peak enhancement by co-injection with authentic standards^31^.

To identify the unknown male compound, preparative-scale GC was performed on a pooled male headspace sample to collect enough material for NMR. An Agilent 7890A GC fitted with an effluent splitter, a Gerstel heated transfer line and a modified Gerstel Preparative Fraction Collector (PFC, Gerstel GmbH & Co. KG, Germany) was used. Chromatography, following the injection of approximately 1 µg aliquots in a volume of 5 µL into a cool-on-column inlet, was done on a HP-1 column (50 m, 0.32 mm ID, 0.52 µm film thickness) using helium as the carrier gas with a flow rate of 2.5 mL/min and an initial oven temperature of 35 °C for 0.5 min, then increased at 5 °C/min to 150 °C, then at 10 °C/min to 230 °C for 10 min. The effluent from the column was split between the flame-ionisation detector (FID) and the PFC in an approximate ratio of 1:120. The transfer line between the oven and the PFC was heated at 250 °C and the PFC at 300 °C. The PFC was modified by connecting the transfer line to a capillary column connector (Supelco, USA). A 5 cm long piece of column (non-polar fused silica, 0.53 mm ID, Supelco, USA) was fitted to the connector when the peak started to appear in the FID trace and disconnected 1.5 min after the peak maximum. The other end of the collection capillary was inserted into 200 µL of redistilled hexane contained in a custom-made vial cooled in solid CO_2_. The vial was made from a Pasteur pipette by heat-sealing at the wider end, thus leaving a very narrow neck, which reduced the risk of loss of compound by evaporation. After collection of the peak, the collection capillary was rinsed into the collection vial with 50 µL of hexane by means of a capillary column connector and a syringe with a fine needle. Each vial was heat-sealed under nitrogen and stored at − 30 °C until all of the material had been fractionated. Finally, the samples were combined, and the hexane solvent was evaporated under a gentle stream of nitrogen to provide a purified sample. The sample was redissolved in deuterated benzene (C_6_D_6_), and 1 mm capillary-probe nuclear magnetic resonance spectroscopy (NMR) analysis was performed using a Bruker Avance 500 MHz spectrometer. The structure was characterised using 1H and gradient COSY (Correlation Spectroscopy), gradient HSQC (Heteronuclear Single Quantum Coherence) and gradient HMBC (Heteronuclear Multiple Bond Correlation) experiments, with the spin systems identified by 1D TOCSY (Total Correlation Spectroscopy). The relative stereochemistry is proposed through 1D nOe (nuclear Overhauser effect spectroscopy) experiments showing correlations through space (see Fig. 4).

### Coupled GC-electroantennography (GC-EAG)

The system has been described in Wadhams^32^. Briefly, an antenna was carefully excised from a live female and suspended between two electrodes, which were made from Ag–AgCl borosilicate glass filled with Ringer solution (without glucose) and connected to silver wire (0.37 mm diam., Biochrom Ltd., Cambridge, UK). The base of the antenna was connected to the grounded electrode, whereas the tip of the outermost lamella was removed to ensure good contact and attached to the recording electrode. A glass tube positioned approximately 5 mm away from the antennal preparation was connected to a stimulus controller (CS-02; Ockenfels Syntech GmbH, Kirchzarten, Germany) and facilitated a continuous flow of charcoal-purified and humidified air towards the antenna at a rate of 10 L/min. Separation of VOCs collected from adult male *L. cervus* was achieved on a GC (6890N; Agilent Technologies, Santa Clara, CA) equipped with a cool on-column injector and an FID, using a 50 m × 0.32 mm ID × 0.52 μm film thickness non-polar HP-1 column. The oven temperature was maintained at 30 °C for 2 min and then programmed at 5 °C/min to 250 °C. The carrier gas was helium. The outputs from the EAG amplifier (UN-06, Syntech) and the FID were monitored simultaneously and analysed on a PC using the Syntech software package for Gas Chromatography -electroantennographic detection (GC-EAG) (v2.3, 09/1997). A peak was defined as EAG-active if in at least three of four coupled runs it evoked an antennal response distinguishable from background noise.

The longifolene was not screened by coupled GC-EAG, because its presence in females had been reported previously^8^ and it was not the primary focus of this study beyond structural and behavioural confirmation.

### Confirming area of volatile release

To confirm the site of volatile release, the cuticle abutting a dense patch of setae on the trochanter of the first pair of legs and the setae in both sexes was examined by scanning and transmission electron microscopy, using a Hitachi S3000N with an accelerating voltage of 20kV. The cuticular area has a high sheen and shape similar to the ovoid patch of setae. Examination of the cuticle on the pronotum beneath the yellow spot area required that the cuticle was excised before hardening of the adult beetle could occur. Therefore, larvae were reared to the pupal stage, collected and placed in soil in a small tank until eclosion, at which point they were placed in a vial of insect fixative (3% gluteraldehyde in 0.1 phosphate buffer). For sample preparation, small areas of cuticle (approximately 1 mm^2^) were excised from the pronotal cuticle, then rinsed in buffer (2 × 10 min), dehydrated through an alcohol series (30%, 50%, 70%, 90%, 100%) for 10 min each, before replacing the 100% ethanol with acetone and critical point-drying, mounting and coating. Specimens were then viewed under various magnifications and examined for the presence of pores. Samples were also prepared for Transmission Electron Microscopy, again using pre-hardened beetles by immersing the tissue in 3% glutaraldehyde in 0.1 M phosphate buffer at pH 7.2 for an hour, followed by 2 × rinsing in buffer for 10 min. The tissue was then placed in post–fixative (1% aqueous osmium tetraoxide) for 2 h, followed by 2 × 10 min of rinsing in water. The tissue was then dehydrated by placing in increasing concentrations of alcohol, up to 100%, as above. For SEM preparation, samples were placed in propylene oxide for 10 min, then overnight in 50:50 propylene oxide and resin, followed by immersion in resin for 6 h. Finally, the specimen was embedded in fresh resin and polymerised at 60 °C for 24 h.

The second and third pairs of legs and abutting cuticle were examined visually for evidence of setae and cuticular adaptations, but since these were not visible, no further sampling was carried out.

### Behavioural assays

All behavioural experiments with live beetles were carried out during their activity period, i.e. dusk, during mid-May to July, in still air arena assays, and the behavioural responses noted. Since numbers of *L. cervus* are naturally low, the experiments took place over three years to ensure adequate replication numbers. The data were analysed using chi-squared and Fisher exact tests to determine differences in response in beetles.

### Determination of cues for mating

To determine whether males use visual or chemical cues to detect females prior to mating, two sets of experiments were carried out with ten individual pairs of mating beetles. On initiating mating, pairs were separated, and the female was placed in turn into a transparent sealed container and an opaque open-ended container. The male was then placed into the choice chamber with the visually or chemically isolated female and his behaviour was observed.

### Response to female and male-produced volatiles

Swab samples collected from male and female beetle ‘yellow spot’ regions were used in behavioural assays on thirty-two male and twenty female beetles to determine activity. Twenty µL (ca. 1-beetle equivalent) of the extracts were placed in clean 0.2 mL PCR tubes, (Eppendorf, UK), a beetle was placed in a central position within the chamber and observed for five minutes to determine its behaviour. All beetles were tested individually with new samples to reduce the chances that a response was due to substances produced in previous tests. The beetles were also exposed to 20 µL of pure diethyl ether, the compound used as a solvent for samples collected on glass swabs. The response of the thirty-two males and twenty females was also tested to 20 µL α-pinene and 20 µL of α-copaene, placed centrally in a clean 0.2 mL PCR tube, as above. The thirty-two males were also tested as above with 20 µL of (−)-β-barbatene and a second, tentatively identified male compound.

To determine male responses to a female-specific compound, later identified as (+)-longifolene, 20 µL of the neat chemical was pipetted into a clean 0.2 mL PCR tube (Eppendorf, UK), which was then placed into a sealed, clear plastic container (10 cm × 10 cm × 6 cm) in the centre of the chamber and the lid closed. The amount of (+)-longifolene emitted this way was estimated, using air entrainment experiments, to be similar to that released by one female beetle ca. 7 cm distance over one hour. The experiment was repeated each time with a new sample of (+)-longifolene to determine whether males can detect it, and therefore females when hidden from view in an opaque container. The twenty female beetles were then tested for their response to (+)-longifolene, as above.

Furthermore, twenty females were placed, in two groups of ten, into choice chambers. A PCR tube containing 20 μL of a neat male-specific compound, later identified as (−)-β-barbatene, was placed centrally in one chamber such that it was approximately equidistant to all females and the beetles observed for signs of sexually receptive behaviour, as described above. The other ten females were placed into an empty holding container to determine whether they produced chemicals eliciting sexually receptive behaviour. Both groups of females were then placed in a choice chamber with males and incidences of males approaching females and initiating copulation recorded.

### Wind tunnel experiments

The response of fifteen males and fifteen females was individually tested to samples taken from the yellow swab area of female beetles in a wind tunnel setup (92 cm × 30 cm × 32 cm). The wind speed was 13–15 cm/s and the temperature 20 °C to mimic a mild summer evening during the flight season of the species. The beetle to be tested was placed downwind of the volatile source at 85 cm and the behavioural response was noted. A positive response was recorded as one in which the beetle moved towards and made contact with the test substance. Beetles that failed to move after 20 min were counted as a negative response. Those that moved in the opposite direction to the stimulus were placed back on the starting point and observed for a further 20 min. Their response was considered negative if they moved away from the stimulus again. Redistilled diethyl ether was used as a control.

### Aerial trap

In 2003 during the flight season, forty aerial traps^8^ were constructed, baited with 20 μL (+)-longifolene placed on cotton wool in a 0.2 mL PCR tube, and distributed to citizen monitors across the UK range of the species**.** Unbaited control traps were sited along with traps containing lures, at a distance of 10 m where possible**.** Traps were placed for maximum of two weeks between 14th and 28th June, as identified as the peak flying season for the species in the UK^6^. For detailed methodology, see Ref.^8^.

### Supplementary Information

Supplementary Figures.

## Data Availability

The datasets generated during and/or analysed during the current study are available via the corresponding authors on reasonable request.
