# Supplementary material for: Novel pheromone-mediated reproductive behaviour in the stag beetle, Lucanus cervus
Source: Sci Rep. 2024 Mar 12;14:6037. doi: 10.1038/s41598-024-55985-8 (PMC10933271; doi:10.1038/s41598-024-55985-8)
Supplement: Supplementary file 1 — Supplementary Figures. [file 41598_2024_55985_MOESM1_ESM.docx]

Supplementary information


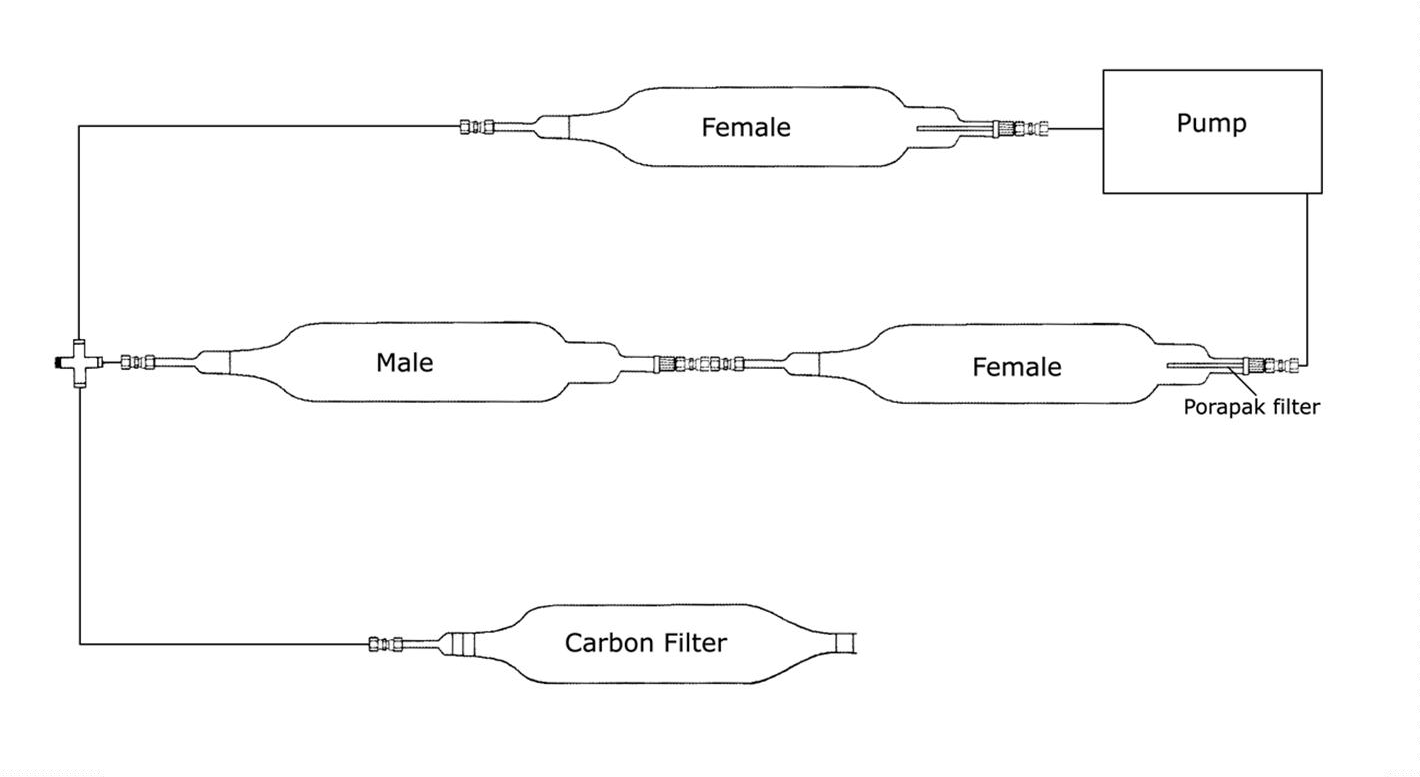


Fig 1 Air entrainment apparatus used to collect volatiles from the head space above addult *Lucanus cervus* onto Porapak

Collection was performed for 22 hours, after which time the volatiles were eluted with redistilled diethyl ether and concentrated by driving through nitrogen gas, then 2 μl were subject to liquid phase gas chromatography followed by mass spectrometry. Gas chromatography was performed on a HP­6890 HP­1 column, length 50 m x 0.32 mm diameter. A run time of 30 minutes was used with the column heated from 30o C to 250oC at a rate of 5oC per minute up to 150oC, then 10oC per minute, from 150­250oC. GC­ Mass spectrometry was carried out in a GC column as above connected to a MAT95XP mass spectrometer. The run time was 49 minutes with the temperature of the column maintained at 30oC for 5 minutes then ramped at 5oC per minute to 250oC. Identification of spectra were made by comparison with the NIST 2002 library.

Longifolene sources: (+) Sigma-Aldrich (CAS Number: 475-20-7, purity %), (-) was isolated from *Scapania undulata* L. (Marchantiophyta: Scapaniaceae) by S. von Reuss/W. Francke (gift to us)

(Schultz AG and Puig S (1985) The intramolecular diene-carbene cycloaddition equivalence and an enantioselective birch reduction-alkylation by the chiral auxiliary approach. Total Synthesis **of** (&)- and (-)-Longifolene. ***J. Org. Chem.* 50,** 915-916)

(-)-ß-barbatene sources: Andersen and Huneck 1973

Andersen NH and Huneck S (1973) Sesquiterpene hydrocarbons of *Bazzania trilobata*. Phytochemistry 12:1818-1819

synthesis: authentic sample from M. Birkett


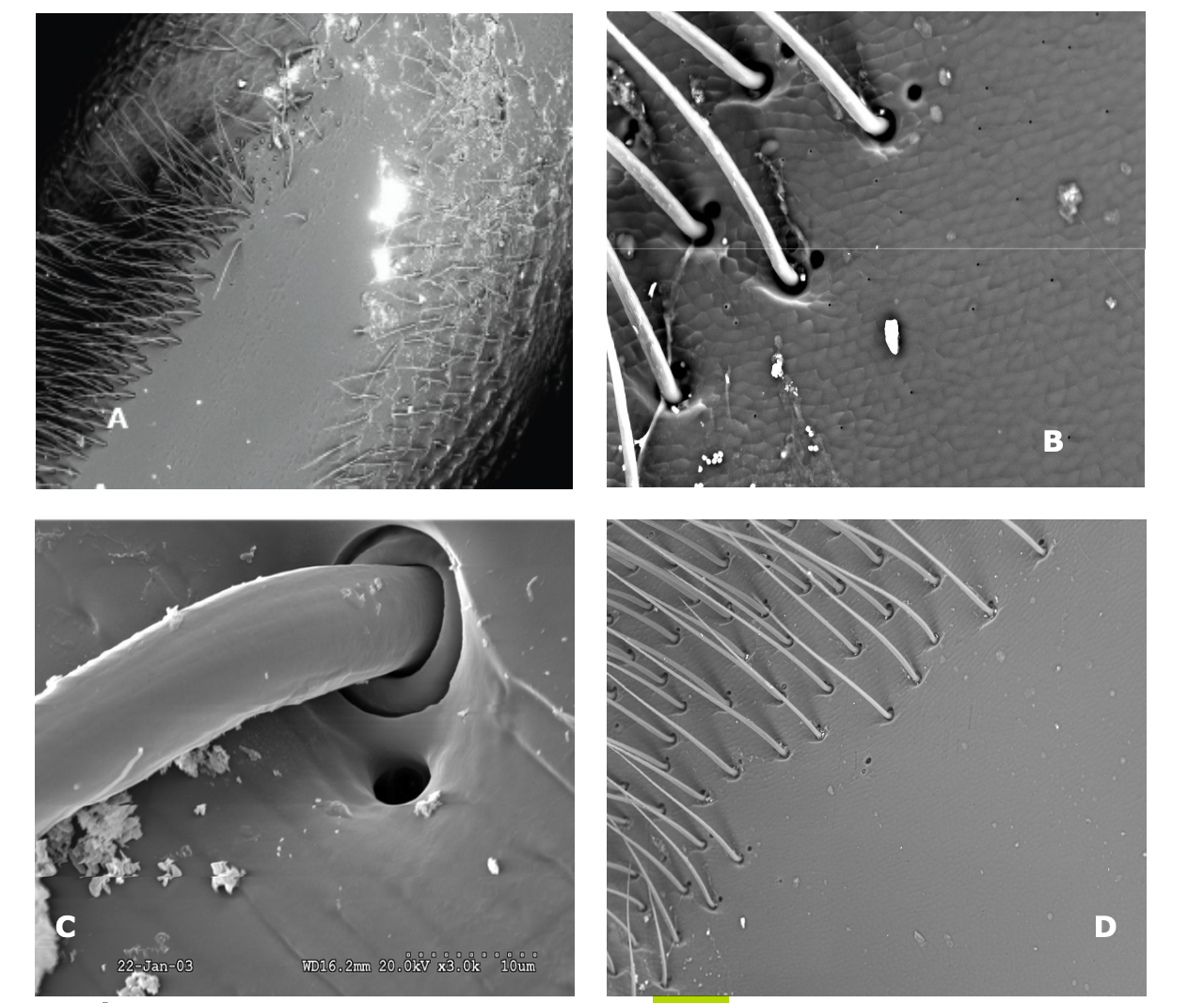


Fig 2 Pronotal cuticle underlying the yellow setae on the first leg, A (Mag=x45).

B (Mag=x250) shows the presence of small holes on the apparently clear cuticular patch within the boundary of the hairs. C (Mag=1700) and D (Mag=x150) show the setae with pores clearly visible at the base.
